# Giant Nonfunctioning Parathyroid Cyst: A Case Report and Review of the Literature

**DOI:** 10.1155/2022/6388749

**Published:** 2022-04-09

**Authors:** Eoin F. Cleere, Mel Corbett, Anne-Marie Quinn, Thavakumar Subramaniam

**Affiliations:** ^1^Department of Otolaryngology-Head and Neck Surgery, Galway University Hospital, Galway, Ireland; ^2^Department of Histopathology, Galway University Hospital, Galway, Ireland

## Abstract

Parathyroid cysts are a rare clinical entity that may arise in the neck or mediastinum. They are more common in women and generally present in the fourth and fifth decades of life. Diagnosis of parathyroid cysts is challenging, and despite thorough radiological and cytological investigation, they are often mistaken for thyroid pathology. Definitive diagnosis is often only confirmed following complete surgical resection and histopathological analysis. We present the case of a woman who was referred to our outpatient clinic with a left-sided neck mass and associated compressive symptoms. Initial examination and investigation appeared consistent with a large thyroid nodule. Following surgical resection, the lesion was found to be a parathyroid cyst. Subsequently, we review the available literature on parathyroid cysts with particular emphasis on the diagnostic challenge they pose to clinicians.

## 1. Introduction

Parathyroid cysts (PCs) are a rare clinical entity with approximately 350 cases reported internationally [1]. PCs pose a diagnostic challenge for clinicians due to varied symptomatology nonspecific signs, or radiologic findings. They are frequently overlooked in the initial differential diagnosis and workup of a neck mass, often requiring excision and histopathological analysis for definitive diagnosis [2]. They may be classified as functioning or nonfunctioning depending on the presence of hyperparathyroidism. The majority of PCs are nonfunctioning and display no biochemical signs of parathyroid hormone dysfunction. Treatment of these nodules typically involves surgical resection. Herein, we describe the case of a patient with a parathyroid cyst and discuss the relevant literature.

## 2. Case Report

We present the case of a 47-year-old female who presented to our head and neck clinic with a long-standing, enlarging left sided anterior neck mass. At presentation, the patient had compressive symptoms, including dysphagia and dyspnoea while supine. Review of symptoms was otherwise noncontributory. She was clinically and biochemically euthyroid. She was a nonsmoker and denied heavy alcohol use. Her medical history was significant for well-controlled asthma with no significant family history.

Clinical examination demonstrated an obvious swelling over the left lobe of her thyroid gland approximately 6.0 × 5.0 cm. On palpation, the mass was firm, nontender, not tethered to any overlying or underlying structures, and had no overlying skin changes. Flexible rhinolaryngoscopy showed no suspicious pathology in the upper aerodigestive tract and symmetric vocal cord mobility. Thyroid ultrasonography was obtained and demonstrated a 6.9 × 5.0 × 4.2 cm benign appearing cystic mass in the left thyroid lobe. All haematological parameters including thyroid function testing and corrected calcium were within the normal range.

**Figure 1:**
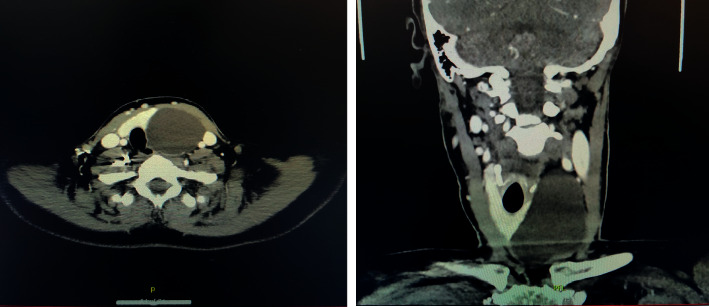
CT neck demonstrating left thyroid cystic mass measuring 7.9 × 5.7 × 5.7 cm displacing left internal jugular vein and left common carotid laterally.

She was subsequently discussed at our institutional endocrine multidisciplinary meeting (MDM). The recommendation from the MDM was to perform left hemithyroidectomy in view of her compressive symptoms. Preoperative fine-needle aspiration cytology was deemed to be unlikely to change the management plan due to the benign appearance of the nodule on ultrasound scan. Before her surgery, the mass had noticeably enlarged, and a subsequent preoperative CT neck (Figure 1) was obtained to assess for interval growth and to aid operative planning.

**Figure 2:**
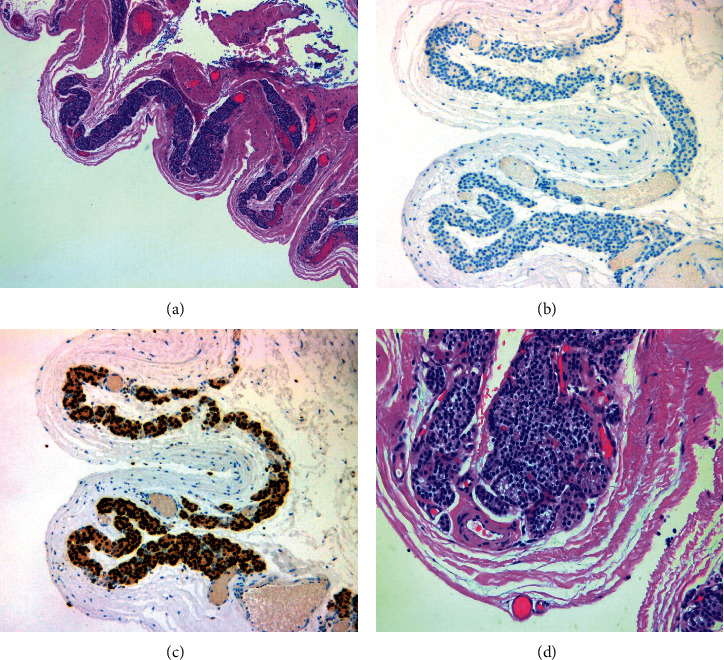
Histological analysis of surgical specimen. (a) Fibroconnective tissue with associated parathyroid tissue lining cystic space. (b) Parathyroid cyst cells staining negative for TTF-1. (c) Parathyroid cyst cells staining positive for GATA-3. (d) Close up visualisation of parathyroid cyst wall.

A transverse anterior neck incision was made within a natural skin crease. Subplatysmal flaps were raised, and infrahyoid muscles retracted laterally. The thyroid gland was visualised and inspected. A large cystic mass was visualised deep to the left lower pole of the thyroid gland that was noted to be inseparable from the thyroid gland. The left recurrent laryngeal nerve was identified, and the mass was excised along with the left thyroid lobe. The mass was completely excised without complication. Histopathological assessment of the mass subsequently confirmed completely excised benign parathyroid cyst (Figure 2). Postoperative haematological parameters were normal including corrected calcium and parathyroid hormone levels which were 2.35 mmol/L and 32 ng/L, respectively. The patient was discharged home well with normal biochemical panel on day 2 postoperatively.

At 6-month follow-up, she remained symptom free with no clinical signs of recurrence and will remain under surveillance at our outpatient department.

## 3. Discussion

PCs are a rare clinical entity. They are usually observed in the fourth and fifth decades of life with a noted female preponderance [3]. They are most commonly found on the left hand side with the inferior parathyroid glands implicated in approximately 70% of cases [1]. They may be divided into functioning PCs (which secrete parathyroid hormone and may be associated with hypercalcemia) and nonfunctioning PCs (which do not secrete parathyroid hormone and are not associated with hypercalcemia). More than 60% of PCs are nonfunctioning and as such less likely to raise suspicion of parathyroid pathology [1].

PCs provide a diagnostic challenge for clinicians. Nonfunctioning PCs classically present as a solitary neck mass, often mimicking a thyroid nodule [4]. Other symptoms such as dysphagia, dysphonia, and dyspnoea may be present. More rarely, PCs may present in the mediastinum [1]. Functioning PCs may present with symptoms of hypercalcemia [5].

Initial radiologic investigation should include ultrasonography of the neck to classify the mass and determine the need for tissue sampling or further diagnostic imaging. Classically, at ultrasound scan, PCs are described as having a nonspecific cystic structure [6]. PCs are usually identified deep to the middle and lower poles of the thyroid gland, and an echogenic border with the thyroid gland may sometimes be observed. PCs retain the original orientation of physiological parathyroid glands and are usually observed to be the largest in the craniocaudal plane [7].

Fine-needle aspiration should be obtained as a diagnostic and occasionally therapeutic intervention. While, in general, clear cystic fluid may not narrow the differential diagnosis of neck masses, a high c-terminal PTH within the fluid can be diagnostic of a PC. Aspirated cystic fluid analysis may show epithelial cells or fibroblasts which may raise false suspicion for malignancy [3]. Computed tomography or magnetic resonance imaging may be indicated for a mediastinal parathyroid cyst or if the above methods fail to adequately diagnose the solitary neck mass [1]. Despite thorough investigation, PCs are frequently misdiagnosed as thyroid cysts until formal histopathological analysis [4, 8]. A case series of PCs report a range of between 1 and 6 cm in maximal diameter that were all separable from the thyroid gland [9]. Giant cysts causing compressive symptoms have been excised en bloc with the thyroid lobe in order to alleviate symptoms and to mitigate the risk of cyst rupture and subsequent parathyromatosis [10]. In our case, MDT recommendation was to proceed with thyroid lobectomy in view of compressive symptoms and suspicion of thyroid pathology. Preoperative diagnosis of a PC may aid the operative planning and subvert the need for thyroid lobectomy.

The pathogenesis of PCs is still unclear, but a number of hypotheses exist [3]. Some hypothesize that PCs may be an embryological remnant from the third or fourth branchial arch with enlargement as a result of colloid accumulation within the cyst. A PC wall often contains a mixture of lymphoid, parathyroid, salivary, adipose, or thymic tissue supporting this theory. Another hypothesis suggests that they occur as a result of parathyroid adenoma degeneration. A third hypothesis proposes that PCs occur following coalescence and enlargement of preexisting parathyroid microcysts. Histologically, PCs have a cyst wall formed by a layer of cuboidal or columnar epithelium which stains positive for glycogen. The presence of parathyroid tissue within the cyst wall is diagnostic [3]. The cyst may be intimately related to thyroid, but it is usually easily separated from it.

A number of treatment options exist for PCs. Surgical resection is historically the most widely cited treatment for parathyroid cysts [1]. Open surgery is associated with morbidity including hypocalcaemia, haemorrhage, and recurrent laryngeal nerve palsy. Nonsurgical management strategies exist, and fine-needle aspiration has been shown to induce cystic regression [11]. Injection of sclerosing agents have also been shown to be effective [12]. These options while potentially appropriate in nonfunctioning PCs do carry the risk of cyst recurrence [13]. Rates of recurrence are difficult to calculate as reporting of PCs is limited to case reports and small series. However, it does appear that recurrence following aspiration is more likely in larger PCs and that recurrence almost never occurs following surgical resection [9]. Surgery is therefore indicated in all functioning PCs to alleviate the symptoms of hypercalcemia, larger PCs, and also recommended in PCs that recur following more conservative approaches.

PCs are a rare pathology requiring a high degree of clinical suspicion for diagnosis. They should be considered as a differential diagnosis for any cystic mass in the neck or mediastinum, particularly if associated with symptoms of hypercalcemia. Definitive treatment of PCs is warranted in all cases, preferably by surgical resection.

## Data Availability

No datasets were generated or analysed during the current study.
